# Klinefelter Syndrome Diagnosis Masked by Opioid Use Disorder

**DOI:** 10.7759/cureus.64870

**Published:** 2024-07-18

**Authors:** Mario Soliman, Karine Delroux-Spalding, Adam Voelckers

**Affiliations:** 1 Family Medicine, University of Pittsburgh Medical Center, Lititz, USA; 2 Family Medicine, WellSpan Community Health Center, York, USA

**Keywords:** sexual dysfunction, hypogonadism, opioid use disorder, substance use disorder, klinefelter syndrome

## Abstract

Klinefelter syndrome (KS) is a chromosomal disorder characterized by the presence of an extra X chromosome in males (47, XXY). Individuals with KS often exhibit a range of physical, cognitive, and behavioral symptoms, including tall stature, gynecomastia, reduced libido, and varying degrees of infertility. A major diagnostic challenge arises when individuals with KS exhibit symptoms that are obscured by comorbid conditions, such as opioid use disorder (OUD). Individuals with OUD or psychiatric disease may exhibit symptoms similar to those of KS. Misattribution of symptoms can lead to delayed or missed diagnosis of Klinefelter's syndrome, underscoring the importance of a thorough evaluation, particularly in the presence of substance use disorders.

In this case report, we illustrate the diagnostic challenges posed by OUD in a 39-year-old male patient with a unique case of undiagnosed KS.

## Introduction

Klinefelter syndrome (KS) occurs when a phenotypic male has two or more X chromosomes. The prevalence of KS is approximately 1 to 2.5 per 1,000 boys and men (0.1%-0.25%), making it the most common human sex chromosome disorder [1-3]. However, only 25% to 50% of individuals with KS are diagnosed during their lifetimes, with most diagnoses occurring in adulthood [1-3]. The median age of diagnosis is 30 years, and 65.2% of cases are diagnosed prenatally [1-3]. The clinical manifestation of KS was initially observed in males who displayed characteristic tall stature, hypogonadism, gynecomastia, and azoospermia [4]. In 1959, researchers pinpointed the genetic cause as the presence of additional X chromosomes [5]. The extra X chromosomes can result in genital anomalies, including hyalinization, fibrosis, and reduced function, typically leading to hypogonadism and infertility [4]. It was not until the middle and later parts of the 20th century that differences in neurocognition associated with KS began to gain recognition [4-6]. Frequently, medical management of KS involves the use of androgen replacement therapy and the application of neuropsychological and adaptive therapies [6]. However, diagnostic gaps, a lack of standardized care, and limited availability and affordability of treatment options limit clinical care [6].

The most prevalent karyotype in KS is 47, XXY, accounting for over 90% of cases. The occurrence of an extra X-chromosome is typically random and often results from errors during meiosis (meiotic nondisjunction) or after fertilization (post-zygotic nondisjunction) [7]. The overall severity of the condition seems to be linked to the quantity of additional X chromosome material present [7-8].

We present a distinctive case of a 39-year-old male patient undiagnosed with KS, whose diagnosis and clinical course were further complicated by opioid use disorder and medication for opioid use disorder.

## Case presentation

A 39-year-old male presented at an outpatient family practice clinic to establish care and evaluation with a complaint of decreased libido for many years. The patient had previously received care at another family practice but wanted to transfer their care to our family practice following a relocation to the area.

The patient has a past medical history of erectile dysfunction and opioid use disorder (OUD). The patient received an OUD diagnosis with heroin being the primary substance of choice since the age of 22. Additionally, the patient has also engaged in the consumption of other substances, including cocaine and methamphetamine, in an attempt to enhance focus and combat persistent fatigue. He also reported using marijuana since the age of eight years old. The patient was receiving buprenorphine/naloxone 16 mg daily and therapy for the treatment of OUD.

The patient reports difficulty in achieving and sustaining erections, leading to significant distress and strain in both current and previous interpersonal relationships. The patient indicated that during a previous stay in a rehabilitation facility, he was advised that his erectile dysfunction and decreased libido were directly related to his heroin addiction. He was informed these symptoms would eventually resolve with the use of phosphodiesterase-5 inhibitors to manage his sexual dysfunction. The patient tried sildenafil 25 mg on an as-needed basis; however, the effectiveness has been variable and inconsistent.

**Figure 1:**
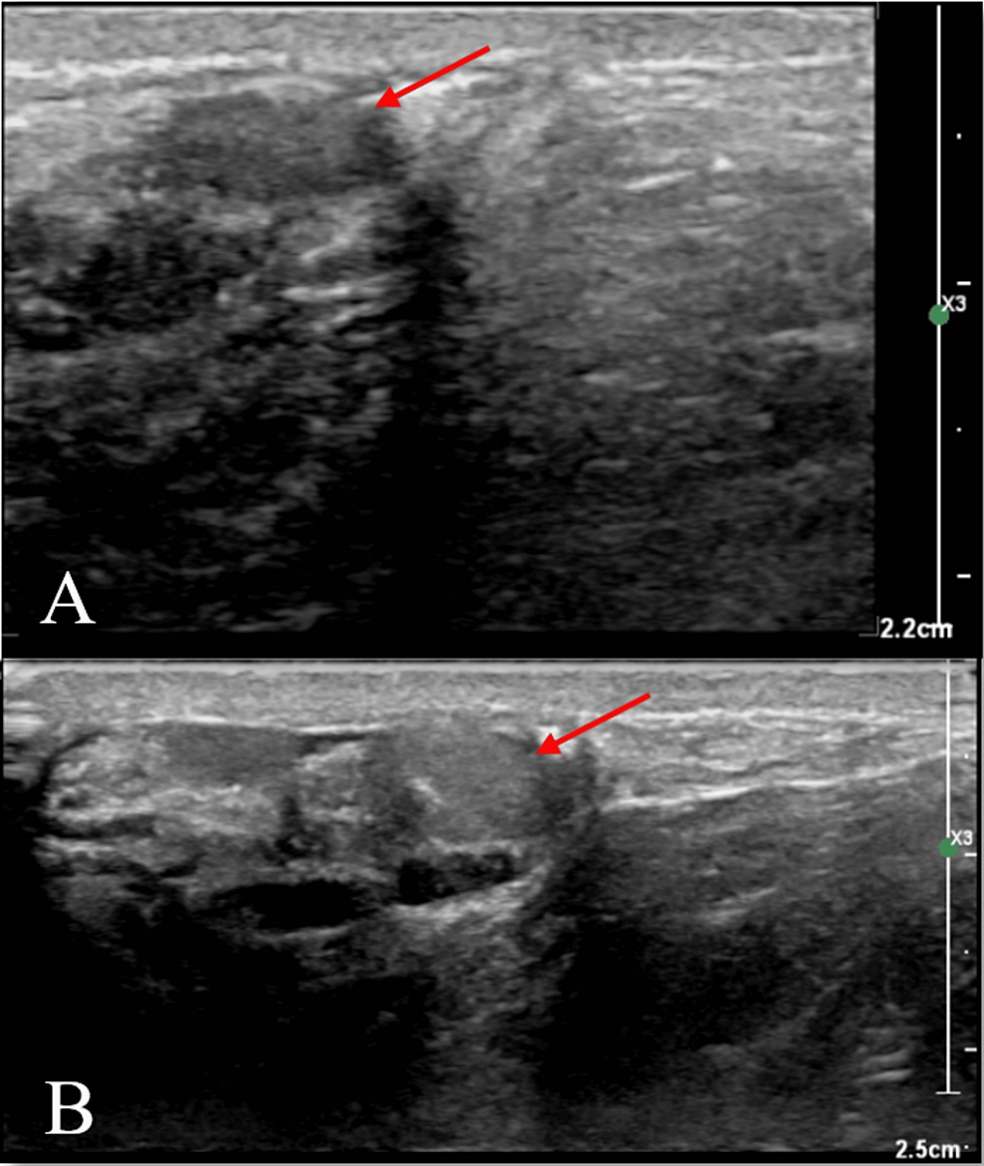
Scrotal ultrasound. (A) The left scrotal ultrasound shows an atrophic left testicle with a measured size of 1.8 cm x 0.7 cm x 1.5 cm and an estimated volume of 1.0 cc. The echogenicity, position, and vascularity of the left testicle and scrotum appear normal.  (B) The right scrotal ultrasound shows an atrophic right testicle with a measured size of 2.0 cm x 0.7 cm x 1.2 cm and an estimated volume of 0.9 cc. The echogenicity, position, and vascularity of the right testicle and scrotum are normal.

During the physical examination, the patient's vital signs were within normal limits. A clinical assessment revealed a female-like body habitus, tall stature, and notably long limbs. On the genitourinary examination, the patient exhibited small testicles and minimal pubic hair. The patient's reported history of sexual dysfunction and the findings from the physical examination prompted a comprehensive evaluation, including assessments of total testosterone, follicle-stimulating hormone (FSH), luteinizing hormone (LH), estradiol, and dehydroepiandrosterone (DHEA) sulfate. The lab results revealed low total testosterone at 50 ng/dL, high FSH at 88.0 mIU/mL, high LH at 29.9 mIU/mL, normal estradiol at 8 pg/ mL, and low DHEA at 95 mcg/ dL (Table 1). A scrotal ultrasound was ordered and revealed symmetrical atrophic testicles with no focal abnormalities (Figure 1). The estimated volume of the right testicle was 0.9 cc, and the left testicle was 1.0 cc per scrotal ultrasound imaging. The average testicular volume for a male in Tanner stage 5 is approximately 20 cc or greater. A chromosomal analysis revealed an abnormal male karyotype with XXY and confirmed the diagnosis of KS. The patient began treatment with biweekly injections of testosterone cypionate depot 100 mg/mL. This therapeutic approach aimed to address his low testosterone levels and, in turn, alleviate his symptoms of fatigue and reduced libido. The patient declined to undergo a semen analysis. Furthermore, genetic counseling was offered to discuss family planning options.

**Table 1:** Summary of laboratory tests conducted, including patient values and corresponding normal ranges.

Laboratory tests	Patient’s value	Normal range
Total testosterone	50 ng/dL	250-1,100 ng/dL
Follicle-stimulating hormone (FSH)	88.0 mIU/mL	1.6-8.0 mIU/mL
Luteinizing hormone (LH)	29.9 mIU/mL	1.5-9.3 mIU/mL
Estradiol	8.0 pg/ mL	≤29 pg/mL
Dehydroepiandrosterone (DHEA) sulfate	95 mcg/ dL	106-464 mcg/dL

## Discussion

This case of KS highlights the importance of a comprehensive evaluation in patients with complex presentations, particularly in patients suffering from OUD. Accurate diagnosis and appropriate management are essential, as the symptoms of these conditions can overlap, potentially leading to delayed diagnosis and treatment.

Opioid-induced androgen deficiency (OPIAD) is now an acknowledged syndrome known for its distinctive features, including decreased testosterone levels, reduced libido, muscle mass reduction, fatigue, and the development of osteopenia [9-10]. It's important to highlight that low levels of both total and free testosterone, along with elevated levels of sex hormone-binding globulin (SHBG), have been documented in patients who use opioids [10-11]. In this case report, it is noteworthy that the patient had been using opioids since the age of 22, which may explain his underlying decreased libido and fatigue. A careful history and physical exam revealed a likely secondary cause of his symptoms and prompted further investigation. Decreased levels of total testosterone can contribute to various symptoms and health issues, including sexual dysfunction and fatigue, as evident in the patient described in this case report [10-11].

Although the patient in our case did not specifically report infertility as a complaint, it is worth noting that various studies have indicated a higher prevalence of oligozoospermia in men using opioids when compared to control groups [12-13]. This observation emphasizes the potential influence of overlapping symptoms between OUD and KS, which may contribute to a delay in the diagnosis and workup for the latter condition. While the exact mechanism behind opioid-induced oligospermia remains unclear, it is well-documented that human spermatozoa express µ-, δ-, and κ-opioid receptors, which are distributed across various regions of the sperm, including the head, middle region, and tail [13]. This suggests that opioids may directly or indirectly affect sperm function and count through their interaction with these receptors [12-13].

It is important to take into consideration KS in patients who have OUD and report sexual dysfunction. Given the overlapping symptoms and common features of these conditions, a comprehensive evaluation becomes imperative to secure an accurate diagnosis and provide suitable management for the patient's overall well-being.

## Conclusions

KS is a chromosomal disorder characterized by an extra X chromosome in males, resulting in a karyotype of 47, XXY. A patient's history of OUD may mask the underlying symptoms of KS, emphasizing the need for careful consideration in such cases. OIAD is now recognized as a distinct syndrome characterized by decreased testosterone levels, reduced libido, muscle mass reduction, fatigue, and osteopenia. Shared symptoms between KS and OUD, which include hypogonadism, reduced libido, and fatigue, can pose a challenge in distinguishing between the two conditions without the necessity of additional diagnostic workup. Additionally, there can be an overlap in laboratory findings, including low total testosterone levels and oligospermia, between KS and OUD. This adds to the complexity of distinguishing between the two conditions based solely on initial test results. Therefore, it is important to consider KS in patients with OUD who present with complaints of sexual dysfunction. A careful history and physical exam can help direct the need for additional testing.
